# The influence of school on whether girls develop eating disorders

**DOI:** 10.1093/ije/dyw037

**Published:** 2016-04-20

**Authors:** Helen Bould, Bianca De Stavola, Cecilia Magnusson, Nadia Micali, Henrik Dal, Jonathan Evans, Christina Dalman, Glyn Lewis

**Affiliations:** ^1^ Department of Psychiatry, University of Oxford, Warneford Hospital, Oxford, UK,; ^2^ Centre for Academic Mental Health, School of Social and Community Medicine, University of Bristol, Bristol, UK; ^3^ Centre for Statistical Methodology, London School of Hygiene and Tropical Medicine, London, UK,; ^4^ Department of Public Health Sciences, Karolinska Institutet, Stockholm, Sweden,; ^5^ Institute of Child Health and; ^6^ Faculty of Brain Sciences, University College London, London, UK

**Keywords:** Eating disorders, school, multilevel, record-linkage, anorexia nervosa, bulimia nervosa, EDNOS

## Abstract

**Background:** Clinical anecdote suggests that rates of eating disorders (ED) vary between schools. Given their high prevalence and mortality, understanding risk factors is important. We hypothesised that rates of ED would vary between schools, and that school proportion of female students and proportion of parents with post-high school education would be associated with ED, after accounting for individual characteristics.

**Method:** Multilevel analysis of register-based, record-linkage data on 55 059 females born in Stockholm County, Sweden, from 1983, finishing high school in 2002-10. Outcome was clinical diagnosis of an ED, or attendance at a specialist ED clinic, aged 16-20 years.

**Results:** The 5-year cumulative incidence of ED diagnosis aged 16-20 years was 2.4%. Accounting for individual risk factors, with each 10% increase in the proportion of girls at a school, the odds ratio for ED was 1.07 (1.01 to 1.13), *P* = 0.018. With each 10% increase in the proportion of children with at least one parent with post-high school education, the odds ratio for ED was 1.14 (1.09 to 1.19), *P* < 0.0001. Predicted probability of an average girl developing an ED was 1.3% at a school with 25% girls where 25% of parents have post-high school education, and 3.3% at a school with 75% girls where 75% of parents have post-high school education.

**Conclusions:** Rates of ED vary between schools; this is not explained by individual characteristics. Girls at schools with high proportions of female students, and students with highly educated parents, have higher odds of ED regardless of individual risk factors.

Key messages

- Eating disorders are more common in some high schools than others.
- Variation in rates of eating disorders between high schools is not explained by any individual student characteristics that the current study was able to assess.
- On average a young woman, regardless of her own background, is more likely to develop an ED if she attends a school with a higher proportion of girls or a higher proportion of children of highly educated parents.

## Introduction

Clinical impression suggests that eating disorders (ED) are more common in some schools than others. We are not aware of any research on this topic, although rates of disordered weight control behaviours do vary between schools. ^1^ ED are particularly common in adolescent girls, ^2^ with a prevalence of 5.7%. ^3^ They have high mortality, with meta-analysis finding a standardised mortality ratio (SMR) of 5.9 in anorexia nervosa (AN), over a mean follow-up period of 14.2 years. ^4^ They are difficult to treat: 10 years after diagnosis, up to 50% still have an ED. ^5^

Schools plays a large part in adolescents’ lives and have been implicated in health problems, including alcohol ^6^^-^^8^ and substance use, ^6^^,^^7^ smoking, ^6^^,^^7^ obesity, ^9^ depression ^10^ and self-harm. ^11^ ED research has not addressed this, although as the proportion of underweight girls in a school increases, so does the likelihood of any individual female student trying to lose weight. ^12^ ‘Composition’ (characteristics of individual students making up each school) differs between schools. Therefore, we would expect a school with a higher proportion of parents with higher education, itself a risk factor for ED, ^13^^-^^15^ to have higher rates of ED. Of more interest is whether there are ‘contextual’ effects of schools that are not explained by individual student characteristics. If such contextual effects exist, possible explanations might be ED being socially contagious, ^16^ or differences between schools in their expectations around eating, exercise and achievement. These might be amenable to preventative intervention.

We hypothesised that incidence of ED in girls would vary between high schools, after accounting for individual students’ characteristics. We hypothesised that higher school proportions of female students, and higher school proportions of parents with post-high school education, would be associated with increased rates of ED. We used a large, contemporary population sample from the Stockholm Youth Cohort, ^17^ Sweden, created by record linkage from health and administrative registers.

## Methods

### Study population

The Stockholm Youth Cohort is a register-based study comprising all 0-17-year-olds resident in Stockholm County from 2001 to 2011( *N* = 735 096), identified through the Register of Total Population (provided by Statistics Sweden). Information on ED and other covariates was obtained for study participants, and their parents, (ascertained via the Multi-Generation Register ^18^ ) through record linkage to health and administrative registers using the national registration number assigned to all residents of Sweden. Information on the ‘Gymnasium’ attended (school for 15-18-year-olds in Sweden, referred to here as ‘high school’) was obtained through Statistics Sweden. We began with a core sample born during or after1983, who left high school in 2002-10 ( *N* = 144 006). The outcome was first ED diagnosis aged 16-20 years.

**Figure 1.**
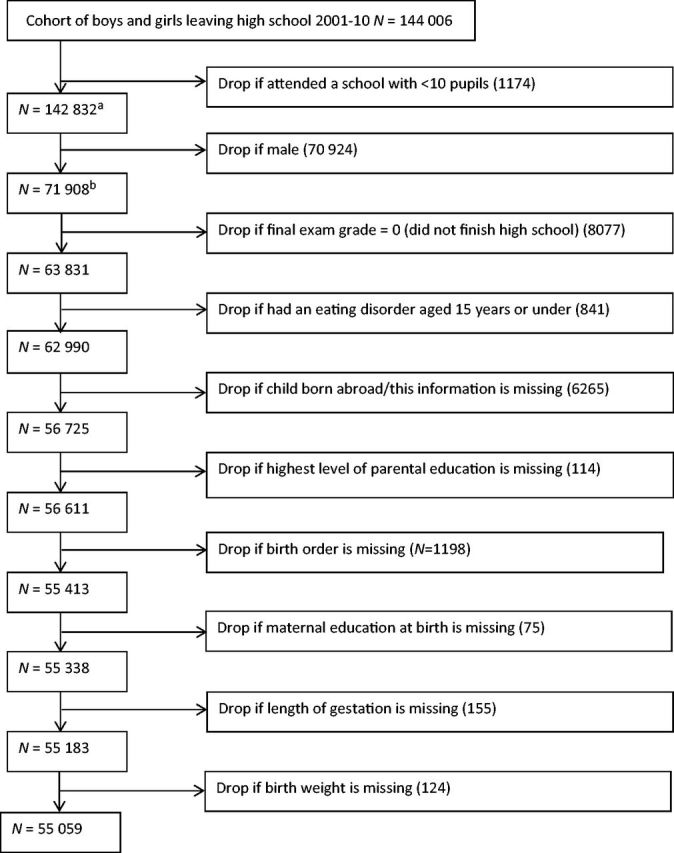
Flow chart of derivation of analytical sample. ^a^ School level variables were created using this sample. ^b^ The school-level variable ‘eating disorders in girls aged 16-20 years’ was calculated from this sample.

Figure 1 shows the final sample derivation. We excluded those attending schools with under 10 students, as these were likely to be due to data entry errors. School environment variables, describing socio-demographic characteristics of the schools, were calculated at this stage from a population of 142 832 subjects. We then excluded boys, as rates of ED vary strikingly between boys and girls, and those scoring zero in their school exit examinations, as this indicated that they did not attend high school for the whole period studied. We excluded children diagnosed with an ED before 16 years of age, as they could not have a first diagnosis of an ED aged 16-20 years. We excluded those born outside Sweden, as we would not have any data on them or their parents at the time of their birth. This left 56 725 girls, attending 409 schools, of whom 55 059 had complete data on all covariates.

### Definition of ED cases

Only first (incident) diagnoses made aged 16-20 years were considered, in order to study the role of school environment. Young people would have been at their high school for 1 year prior to a diagnosis. Diagnoses occurring up to 2 years after leaving school were included, to allow for delayed diagnosis.

Participants were identified as having a diagnosis of an ED if they were given an ICD code indicating diagnosis of any ED [any F50 code in ICD-10 (AN, atypical AN, BN, atypical BN, overeating or vomiting associated with other psychological disturbances, other ED, ED unspecified); 307.1 (AN)/307.5 (BN, ED not otherwise specified) in ICD-9], or the equivalent DSM-IV codes, by a specialist clinician as an inpatient or outpatient during follow-up. Cases were also identified through attendance at a specialist ED clinics, as we judged it very likely that these individuals had an ED. Previous work in this cohort has shown that findings are unchanged in sensitivity analyses where outcome is restricted to a recorded diagnosis of ED or attending a specialist ED clinic at least three times. ^13^ Sources for identifying ED cases were: (i) Clinical Database for Child and Adolescent Psychiatry in Stockholm (DSM-IV diagnoses from child and adolescent psychiatric services, 2001 onwards); (ii) Stockholm County Council Health Service Use Register (VAL) (clinic attended, 1997 onwards); (iii) Stockholm Adult Psychiatric Care Register (DSM-IV diagnoses until 2004, ICD-10 diagnoses 2005 onwards, from all publicly-run adult psychiatric care services within Stockholm County); (iv) National Patient Register (ICD diagnoses for all psychiatric inpatients, 1973 onwards).

### Other variables

Record linkage enabled us to obtain data on maternal age at childbirth, parental level of education and disposable income, country of birth, examination scores, family type at birth of child (single- or two-parent family) and number of siblings at birth of child. Parental mental health variables were obtained from any psychiatric diagnosis from sources ii, iii and iv described for ED diagnoses above, and also primary care records. Gestational age and birthweight were ascertained from the Medical Birth Register. ^19^ School environment variables were generated from data on 142 832 male and female subjects, by calculating the proportion of the students at each school in the sample who were female, who had at least one parent with post-high school education and so on (see Table 1 ). The Stockholm Regional Ethical Review Board approved the study.

**Table 1.** Description of Individuals and schools

	**Variables**	** Whole sample ( *N* varies by covariate) %, *N* /total *N***	** Complete case sample (N = 55 059) %, *N***
Individual-level variables	Eating disorder aged 16-20 years	2.4 (1354/56 725)	2.4 (1306)
	Final exam score in the top 20%	30.5 (17 289/56 725)	30.5 (16 798)
	Disposable income in the top 20% for the whole of Sweden	34.7 (19 693/56 723)	34.8 (19 183)
	One or both parents with post-high school education	56.7 (32 090/56 611)	56.7 (31 193)
	Mother with post-high school education	36.7 (20799/56 627)	36.7 (20 211)
	One or both parents born outside Sweden	26.9 (15 237/56 725)	26.5 (14 584)
	Mother has history of psychiatric illness	31.4 (17 804/56 725)	31.3 (17 244)
	Father has a history of psychiatric illness	20.5 (11650/56 725)	20.5 (11 305)
	One or both parents have a history of psychiatric illness	43.5 (24 685/56 725)	43.5 (23 923)
	Maternal age (mean, SD)	29.1 (5.1)	29.1 (5.1)
	Length of gestation (weeks) (mean, SD)	39.4 (1.8)	39.4 (1.8)
	Birthweight (kg) (mean, SD)	3.4 (0.5)	3.4 (0.5)
School-level variables ^a^	Number of students per school (median %, interquartile range)	1310 (671–2281)	1310 (671-2281)
	Eating disorder in girls aged 16-20 years (median %, interquartile range)	2.4 (1.5-3.0)	2.4 (1.6-3.0)
	Female(mean %, SD)	55.0 (13.1)	55.0 (13.1)
	Final exam score in the top 20% (median %, interquartile range)	18.1 (11.6–30.3)	18.2 (11.6–30.4)
	Disposable income in the top 20% for Sweden (mean %, SD)	32.4 (12.2)	32.5 (12.2)
	One or both parents with post-high school education (mean %, SD)	57.2 (17.3)	57.2 (17.3)
	Mother with post-high school education (mean %, SD)	37.5 (15.9)	37.5 (15.9)
	One or both parents born outside Sweden (median %, interquartile range)	24.6 (19.1–30.4)	24.6 (19.1–30.4)
	Child born abroad (median %, interquartile range)	5.1 (3.3-7.7)	5.1 (3.3–7.7)

SD, standard deviation.

^a^ School level variables were calculated using all available data from 142 832 individuals (see flow chart), with the exception of ‘eating disorders in girls aged 16-20 years’, which was calculated using girls only (71 908 girls).

### Analysis

We described the whole sample and compared it with the sample with complete data on covariates (‘complete case sample’) ( Table 1 ). To examine clustering of ED diagnoses in pupils from the same schools, we used mixed effects logistic regression models ^20^ to estimate odds ratios (ORs) for ED in relation to individual- and school-level variables, using the command ‘xtmixed’ in Stata 13, treating students as elementary units and schools as clustering units. These models also allowed calculation of the intraclass correlation coefficient (ICC) corresponding to each fitted model. When the model is fitted without explanatory variables, the ICC represents the percentage of total variation in (log) odds of ED that is due to differences between schools (‘between-school heterogeneity’). Some of these differences can be explained by individual student-level variables, such parental education. We therefore also calculated ICCs for models including individual student variables, first separately and then jointly (choosing those which separately had the greatest effect on the ICC). When school-level variables are included in the model, the ICC represents the percentage of residual variation that is due to between-school heterogeneity ( Table 2 ).

**Table 2.** Percentage of unexplained variation in incidence of eating disorders at the school level, adjusting for individual-level variables (females only). For unadjusted models, the ICC represents the % of total variation due to between-school heterogeneity

	ICC (% of residual variance due to between-school heterogeneity) (95% CI)
**I. Unadjusted models**	
i. Whole sample ( *N* = 56 725)	4.5 (2.8 to 7.1)
ii. Complete case sample ( *N* = 55 059)	4.4 (2.8 to 7.1)
**II. Models adjusted for separate individual-level variables**	
i. Disposable income ^a^	4.1 (2.5 to 6.7)
ii. Highest level of education of either parent ^b^	3.1 (1.7 to 5.5)
iii. Mother’s level of education ^b^	3.5 (2.0 to 6.0)
iv. Child’s year of birth	4.4 (2.0 to7.0)
v. Mother’s age at birth of child (years)	4.1 (2.5 to6.7)
vi. Either parent born outside Sweden	4.3 (2.7 to 6.9)
vii. History of psychiatric illness in either parent	4.6 (2.9 to 7.3)
viii. Birth order	4.4 (2.7 to7.0)
ix. Length of gestation	4.4 (2.8 to 7.1)
x. Birthweight (kg)	4.4 (2.7 to7.0)
**III. Model adjusted for multiple individual-level variables**	
i. Parental level of education, ^b^ history of psychiatric illness in either parent, maternal age, disposable income, ^a^ either parent born outside Sweden	2.9 (1.6 to 5.3)
**IV. Models adjusted for separate school-level variables (one variable at a time)**	
i. Female	4.4 (2.7 to 7.0)
ii. Born outside Sweden	3.8 (2.3 to 6.3)
iii. Final exam score in the top 20%	2.4 (1.2 to 4.7)
iv. Disposable income in the top 20%	2.0 (0.9 to 4.3)
v. One or both parents with post-high school education	1.2 (0.4 to 3.5)
vi. One or both parents born outside Sweden	4.0 (2.4 to 6.6)
**V. Models adjusted for separate school-level variables (one variable at a time) and individual-level variables** ^c^	
i. Female	2.8 (1.5 to 5.2)
ii. Born outside Sweden	2.5 (1.3 to 4.8)
iii. Final exam score in the top 20%	1.8 (0.8 to 4.1)
iv. Disposable income in the top 20%	1.6 (0.6 to 3.9)
v. One or both parents with post-high school education	1.2 (0.4 to 3.5)
vi. One or both parents born outside Sweden	2.7 (1.4 to 5.1)

^a^ In quintiles, treated as an ordinal variable.

^b^ Three levels [1 = did not attend high school, 2 = high school (school for 15-18 year olds), 3 = post-high school, treated as an ordinal variable].

^c^ Individual-level variables: child’s year of birth, history of psychiatric illness in either parent, highest level of parental education (3 level, ordinal), maternal age at birth of child, disposable income (5 level, ordinal) and whether either parent was born outside Sweden.

**Figure 2.**
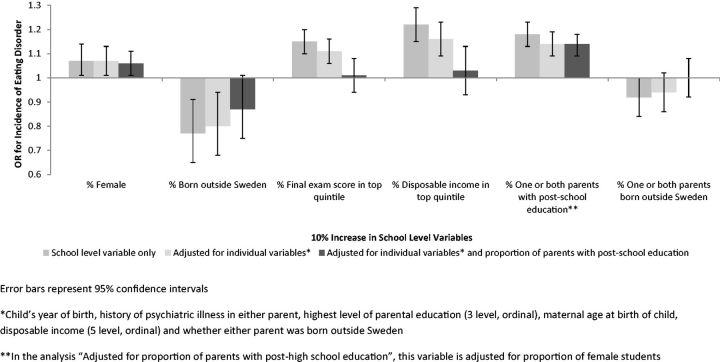
Odds Ratio for 5-year cumulative incidence of eating disorder diagnosis for a 10% increase in school variables.

Adding school environment variables (such as percentage of girls attending the school) to the models allowed us to estimate ORs for ED for different school environment factors and to calculate relative contributions of these variables to between-school variation. The sequence of models first examines the effect of adding one school environment variable at a time (with and without controlling for individual student-level variables), and then of each variable together with the proportion of students at the school with at least one parent with post-high school education ( Figure 2 ).

We also investigated whether there was an interaction between the proportion of children at a school with at least one parent with post-high school education, and the highest parental educational level of each individual student. We created an interaction term for this combination in order to test the hypothesis that individuals were at higher risk of ED if they were different from their peers (i.e. a student with parents with a lower level of education in a school with a high proportion of highly educated parents, and vice versa).

One explanation for differences in proportions of students identified with an ED in different schools is that parents with post-high school education may be more aware of available services. To test this explanation, we used the complete model described above to investigate whether being seen in Child and Adolescent Mental Health Services (CAMHS) for any mental health problem was associated with the proportion of students who have one or both parents with post-high school education.

A final model including school proportion of children with at least one parent with post-high school education, school proportion of girls, and all individual-level variables, was used to predict the probability of ED for an average girl in different school settings ( Table 3 ).

**Table 3.** Probabilities (expressed as percentages) of being diagnosed with an eating disorder for an average girl in different school environments

% Parents with post-high school education	% Girls	Predicted probability of ED ^a^ (%)
25	25	1.3
25	50	1.5
25	75	1.8
25	100	2.0
50	25	1.8
50	50	2.1
50	75	2.4
50	100	2.8
75	25	2.5
75	50	2.8
75	75	3.3

^a^ Probabilities predicted from the final mixed effects logistic regression model that included all individual-level variables. Variables other than % parents with post-high school education and % girls were set at the mean or modal values as follows: one or both parents with post-high school education; no parental psychiatric history; maternal age at birth of child = 29; disposable income in the top quintile; parents both born in Sweden.

All analyses were conducted using Stata 13.

## Results

### Descriptive data

The sample came from 409 schools. Individuals in the complete case sample had slightly fewer parents born abroad and slightly higher disposable income than the whole sample. School environment variables were very similar for whole and complete case samples. 2.4% of the 55 059 girls in the complete case sample were diagnosed with an ED aged 16-20 years. Of these, 22% had a diagnosis of AN, 14% a diagnosis of BN, and the remainder had an ED inferred from attendance at a specialist ED clinic; 94% of the cohort remained in Stockholm county the year after leaving high school.

### School environment and rates of ED

Of the total variation in odds of ED, 4.4% [95% confidence interval (CI) 2.8 to 7.1] was due to differences between schools. The individual student characteristics with the greatest impact on this variation were parental level of education, parental psychiatric history, maternal age, disposable income and having a parent born outside Sweden. When all these individual characteristics were included in the model, the variation in the odds of ED due to variation between schools reduced to 2.9% (95% CI 1.6 to 5.3).

We examined associations between school characteristics and odds of ED. After adjusting for individual risk factors, with each 10% increase in the proportion of girls at a school, the odds ratio for ED was 1.07 (1.01 to 1.13), *P* = 0.018. With each 10% increase in the proportion of children with at least one parent with post-high school education, the odds ratio for ED was 1.14 (1.09 to 1.19), *P* < 0.0001. Attending a school with a greater proportion of students achieving examination results in the top 20% of the population, or with disposable income levels in the top 20% of the population, was also associated with increased odds of being diagnosed with an ED. Attending a school with a higher proportion of students born outside Sweden was associated with decreased odds of developing an ED. Adjustment for school proportion of parents with post-high school education substantially reduced the ORs of all school-level variables except percentage of females [OR 1.06 (95% CI 1.01 to 1.11%), *P* = 0.026; Figure 2 ].

After adjusting for each of the other school characteristics in turn, a 10% increase in the proportion of parents with post-high school education remained associated with an increase in the odds of ED [e.g. 1.14 (1.09 to 1.18), *P* < 0.0001 after adjusting for proportion of girls].

We found no evidence of an association between school proportion of parents with post-high school education and referral to CAMHS for any mental health problem [OR 1.00 (95% CI 0.98, 1.05), *P* = 0.739]; nor was there evidence of other school-level variables being associated with referrals to CAMHS. There was no evidence of interaction between individual- and school-level parental education ( *P* = 0.44).

Table 3 shows predicted probabilities of an average girl developing an ED in different school environments, based on the final additive model holding all individual-level variables at the mean or modal values. The predicted probability of developing an ED is: 1.3% for pupils in a school with 25% girls, where 25% of the children have at least one parent with post-high school education; and 3.3% in a school with 75% girls where 75% of children have at least one parent with post-high school education.

## Discussion

### Main Findings

Diagnosed ED are more common in girls at some schools than others, and this is not explained by the individual characteristics of the students. Aspects of school environment are associated with ED. On average a young woman, regardless of her own background, is more likely to develop an ED if she attends a school with a higher proportion of girls or of children of highly educated parents.

### Strengths and limitations

As far as we are aware, this is the first study to investigate whether rates of diagnosed ED among girls vary between schools. Methodological strengths include its total population design, large sample size and prospective information on potentially confounding variables. Register-based studies ^13^^-^^15^^,^^21^^,^^22^ contribute to our understanding of ED aetiology by enabling us to study very large numbers of people with diagnosed ED. Limitations of the present study include the lack of information on all individual characteristics which may predispose to ED. Second, not all individuals with ED seek treatment, and those that do may be seen in primary care or in private services, for whom we do not have data, so we are likely to have underestimated rates. In the USA, only 3-28% of adolescents with an ED report receiving specific treatment for weight or eating problems ^23^ ; numbers may be higher in Sweden, where there is a comprehensive, government-provided health service, but barriers to treatment such as stigma, denial, and lack of public awareness may still play a role. Rates of diagnosed ED may vary between schools if those with greater numbers of educated parents are better at identifying ED and directing students towards help. We found no evidence of an association between higher proportions of educated parents and being seen within Child and Adolescent Mental Health Services, but barriers to treatment-seeking may be different for eating disorders than for other psychiatric disorders.

We did not study AN, BN or eating disorder not otherwise specified in separate analyses because of limited statistical power, partly because some sources do not differentiate between subtypes. Results cannot be generalized to immigrant populations, as those born abroad were excluded due to a lack of data.

### Comparison with previous findings

Variation in rates of ED due to differences between schools was similar to the variation of disordered weight control behaviours between schools in a US sample, ^1^ although we found that differences between schools persisted after adjusting for individual variables. Differences may be due to our study being much larger, the nature of our (treatment-seeking) sample, or differences between disordered weight control behaviours and diagnosed ED.

### Possible mechanisms

The primary purpose of our analysis was to demonstrate an association between school environment factors and eating disorders, but future research may consider whether the associations we have observed may be causal, rather than explained by unmeasured confounding or other sources of bias. Possible ways in which school environment might in that case contribute to the development of ED include those around the idea of ED being contagious, and those relating to differences in school environments, for example in schools’ expectations around achievement.

Schools with more girls, or more highly educated parents, are likely to have some students with ED because female gender and parental education are risk factors for ED. However, differences between schools may be inflated if ED are contagious and can spread within schools. There are various mechanisms whereby ED may be contagious. Social comparison theory ^16^ suggests that comparing one’s own body negatively with those of others leads to body dissatisfaction (a known risk factor for ED). ^24^ Body size perceived as most normal and attractive is altered through exposure to women of different sizes, both in the laboratory ^25^^,^^26^ and in real life. ^27^ Rates of body dissatisfaction are higher in areas with lower mean population body mass index (BMI), and vice versa. ^28^ In a school with a lower mean BMI (perhaps due to some students having ED), students might perceive themselves as larger, leading to higher levels of body dissatisfaction and more ED. The finding that in schools with greater proportions of underweight girls, individual girls are more likely to try to lose weight, ^12^ supports this idea.

Students may also ‘learn’ eating disordered behaviours from witnessing others. Research suggests that body image concern, ^29^ extreme weight loss behaviours ^29^^,^^30^ and bingeing ^31^ are influenced by friendship group.

All schools have unique environments. They may vary in food provided, whether students bring a packed lunch or can easily leave at lunchtimes, whether students are expected to take part in extra-curricular activities during their lunch break, and presence and contents of vending machines. These variations are associated with differences in behaviour: number of snacks purchased from vending machines increases with number of vending machines in a school, ^32^ and pupils consume more fruit and vegetables in schools with salad bars. ^33^ More subtly, schools have distinctive cultures which may influence student beliefs, attitudes and behaviour regarding eating, exercise and appearance. Schools with more students from more educated families may have higher aspirations and exert greater demands on their students. This may encourage perfectionism, which is strongly associated with ED, ^34^ as girls strive to attain the goal of the ‘thin ideal’. ^35^ Thus an aspirational school culture may inadvertently lead to increased rates of ED. Compared with their co-educated peers, girls in single-sex schools view achievement as more important, and are more likely to associate intelligence and professional success with being thinner. ^36^

Another possible explanation is that differences between schools could be due to differences in treatment-seeking, rather than differences in rates of ED per se.

### Implications

Swedish gender equality laws mean all schools must admit both sexes, although occasionally student choice may lead to some schools being single-sex. Nevertheless, we believe that these results are likely to generalize to other countries. We do not know whether the proportion of girls and proportion of highly educated parents in a school cause an increase in the number of diagnosed eating disorders, but these results suggest that female students at fee-paying or selective schools are more likely to have a diagnosed ED, particularly if the schools are single-sex. If future research finds school environment to be causal in the development of ED, preventative interventions aimed at schools themselves, for example, training teachers, may be valuable in schools with high rates of ED. If differences are in fact due to differences in rates of treatment-seeking, schools with lower rates may need support in raising awareness or reducing stigmatization of eating disorders.

## Funding

This work was supported by the Wellcome Trust Institutional Strategic Support Fund in the form of an Elizabeth Blackwell Clinical Primer (to HB); and Stockholm County Council (to CM, CD, HD).

*Conflict of interest* : The authors have no conflicts of interest to declare.
